# *Ex vivo* evaluation of polyethylene cable compared to stainless steel cerclage wire in a canine fracture model

**DOI:** 10.3389/fvets.2025.1613295

**Published:** 2025-07-16

**Authors:** Seila Day, Steven Elder, Cooper Brookshire, Michael H. Jaffe

**Affiliations:** ^1^Department of Clinical Sciences, Mississippi State University College of Veterinary Medicine, Mississippi State, MS, United States; ^2^Bagley College of Engineering, Agricultural and Biological Engineering, Mississippi State University, Mississippi State, MS, United States

**Keywords:** UHMWPE (ultra-high molecular weight polyethylene), cerclage wire, fracture repair, canine, biomechanical, femur, orthopedic cable

## Abstract

**Introduction:**

The objective of this study was to compare the biomechanical stability of an ultra-high molecular weight polyethylene (UHMWPE) orthopedic cable system to stainless steel cerclage wire (SSW) fixation in cyclic four-point bending in a cadaveric fracture model.

**Materials and methods:**

A long oblique osteotomy model was employed using paired canine cadaveric femurs. The osteotomies were stabilized with either three UHMWPE cables (*n* = 10) or three 18 gauge stainless steel loop cerclage wires (*n* = 10). Cyclic testing was performed by applying increasing force at 2 Hz until construct failure, defined as ≥2 mm of actuator displacement. Data analyzed included cycles to failure, load at failure, and dynamic stiffness.

**Results:**

There was no statistically significant difference in any of the outcomes tested between constructs. Visible loosening was noted in all loop cerclage constructs. No visible loosening of the UHMWPE cable was noted.

**Discussion:**

The results suggest that the UHMWPE cable’s resistance to failure was comparable to SSW in four-point bending. Additional biomechanical testing would be needed to assess for statistical significance as well as performance in torsion and compression or with adjunctive fixation methods. Future clinical studies in veterinary applications are needed to assess how the UHMWPE cable performs *in vivo*.

## Introduction

Cerclage wire is a commonly used orthopedic implant in veterinary surgery as an adjunct to other fixation modalities providing interfragmentary compression of bone fragments. Monofilament stainless steel cerclage wire is versatile and can be utilized in many ways including in repair of long oblique fractures in conjunction with an intramedullary pin or bone plate, periprosthetic fractures following total hip arthroplasty, or in combination with a pin to form a tension band for treatment of avulsion fractures. Monofilament stainless steel wire (SSW) can be secured using several techniques such as the twist method, bent eyelet wire method, double-loop cerclage method, double-wrap cerclage method, and loop/twist technique and its mechanical properties have been extensively studied (1–8).

Although SSW is relatively mechanically strong and economic, metallic cerclage has been linked to post-operative complications including soft tissue irritation, premature breakage and loosening, loss of fixation, infection, and implant associated pain. Many of these complications lead to reoperation (9–13). Non-metallic cable and suture has been shown to be biomechanically similar in strength to metallic wire fixation and human medical literature reviews and retrospective studies suggest its use may lead to fewer complications (including wire loosening, fixation loss, and delayed healing), making noon-metallic cable seem promising as an alternative to metallic cerclage fixation (9–12). One such cable emerging in the market combines a nylon core with a jacket of braided ultra-high-molecular-weight polyethylene (UHMWPE) polymer fibers and is secured with a titanium alloy locking clasp.^1^

The veterinary literature is limited to few biomechanical studies directly comparing the use of non-metallic orthopedic systems compared to SSW. Rothaug et al. and Hwang and Kim investigated the use of non-metallic cable (Secure Strand and FiberWire respectively) in cadaveric models and their findings suggest non-metallic cable to be a viable alternative method of fixation (14, 15). To the authors’ knowledge, no veterinary studies have been reported testing the combined nylon and UHMWPE cable system against other cerclage materials under cyclic load. There are also no reports found demonstrating this cable system under four-point bending, compression, and torsional testing in either a simulated fracture model or a cadaveric fracture model.

The aim of this study was to investigate the biomechanical stability of UHMWPE cable compared to stainless steel cerclage wire in a canine cadaveric long-oblique femoral fracture model. The study employed cyclic four-point bending until failure to assess performance differences in cycles to failure, load at failure, and dynamic stiffness. As a pilot study, the primary objective was to determine variance and effect size to guide future research and sample size calculations. The secondary objective was to identify any statistically significant differences between the two fixation methods and assess modes of failure. The authors hypothesized that the UHMWPE cable system would have superior biomechanical stability compared to stainless steel wire under these testing conditions.

## Materials and methods

This study used 20 paired cadaveric canine femurs (10 matched femurs for each fixation method), chosen to minimize confounders and effect modifiers. The matched design with a standardized bone size was intended to enhance the reliability of our results by reducing variability unrelated to the fixation methods themselves. While the primary aim of this pilot study was to assess variance and effect size to inform future research, we estimated that a sample size of 10 femurs per group would be reasonable for descriptive statistics. Furthermore, should there be a standard deviation of 2000 cycles to failure per group and a difference of 2000 cycles between groups, this sample size would provide sufficient power (90% at *α* = 0.05) to detect a statistically significant difference between the UHMWPE cable and SSW constructs.

Rather than simulating fracture repair, the study was designed to isolate the differences in mechanical stability of the implants by utilizing a standardized long-oblique cadaveric model under cyclic four-point bending. Loop cerclage was chosen over the standard twist method because of its relative ease of application, higher loop tension, and comparable load to loosen versus cerclage placed using the twist technique (2). Femurs were used as a model due to the uniform shape of the diaphysis and frequent use of cerclage in clinical fractures affecting this bone. Four-point bending was chosen as it applies a uniform bending moment across the specimen and represents one of the principal physiologic forces acting on a fracture (5, 16, 17). The force applied to the constructs increased over the testing period beyond typical physiologic load generated by a walking dog with hopes of highlighting mechanical differences of the implants and assess mode of failure under these supra-physiologic conditions.

### Specimen preparation

Twenty paired femurs (ten dogs) weighing between 18 kg and 29 kg were harvested from humanely euthanized adult dogs acquired from a local Humane Society and were stored frozen at −20°C. The dogs were euthanized for reasons unrelated to the study and their limbs were grossly inspected to confirm full maturity and for any obvious injuries that would preclude their use. After thawing for 24 h at room temperature all soft tissues were removed, and a standardized long oblique fracture model was created. The ends of the bones were embedded in polyurethane casting resin (Fabri-Cast 50, Hardness 75-D), and a band saw (0.5 mm in width) was used to create a cranial-proximal to caudal-distal long oblique osteotomy midway through each bone’s diaphysis. The angle of the osteotomy created was calculated based on diameter of each individual bone to allow 3 cm along the diaphysis for placement of three SSW or UHMWPE cables to mimic appropriate placement of implants for a long oblique fracture repair.

### Fixation

The femoral osteotomies within each pair were stabilized using one of two techniques, either three 18 gauge (1 mm) loop cerclage wires or three UHMWPE cables, (1.5 mm) were placed across the osteotomy at 1 cm intervals and 0.5 cm from the cut ends of the osteotomy in accordance with AO principles of cerclage wire placement (8). Each UHMWPE cable was tensioned to 120 lbs. using the manufacturer’s calibrated tensioning instrument. Loop cerclage wires were placed using AO standard surgical techniques by a single experienced surgeon to reduce variability in factors such as wire tension that can arise with multiple operators. Fixation method used for the left and right femurs were randomly assigned to the first pair of femurs by coin flip, then alternated between right and left for each subsequent tested pair.

### Mechanical testing

**Figure 1:**
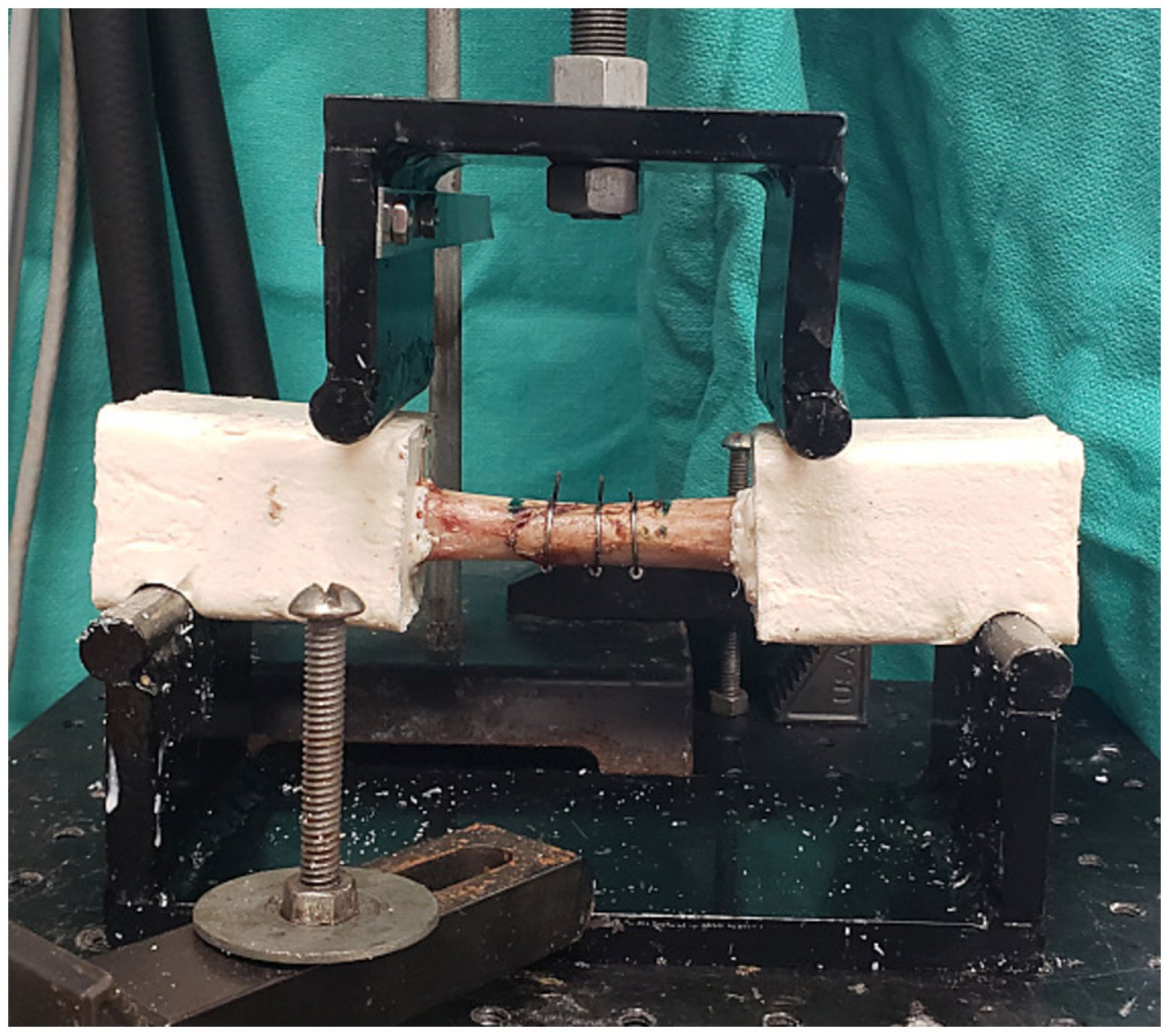
Setup for mechanical testing.

The models were tested cyclically under four-point bending using a servohydraulic testing device (Bionix 858: MTS Corporation, Eden Prairie, Minnesota, United States). Notches placed in the resin engaged the supports to prevent axial separation of the two bone ends during testing. The starting position was established by manually bringing the loading noses into contact with the specimen and then applying displacement until the load reached 30 N. At this point, the machine was transferred to load control for cyclic testing. Sinusoidal compression at 2 Hz was applied to create a cyclic medial to lateral bending moment varying from approximately 1.5 to 4 N-cm (constant moment between the loading noses). The upper force increased by 100 N every 1,000 cycles and the lower force was kept consistent at 10 N. Failure was considered as the point at which position of the loading noses under maximum load was ≥ 2 mm from the starting point. Number of cycles to failure, dynamic stiffness (N/mm), and load at failure (N) was determined for all constructs (Figure 1).

### Statistical analysis

Statistical analyses were performed using SPSS (Version 29.0.2.0). To assess whether the data met the assumptions of normality, the Shapiro–Wilk test and visual inspection of a Q-Q plot were conducted. The results indicated that the differences between the two groups for all variables were approximately normally distributed (cycle to failure Shapiro–Wilk: W = 0.891, *p* = 0.174; load at failure Shapiro–Wilk: W = 0.889, *p* = 0.164; dynamic stiffness Shapiro–Wilk: W = 0.855, *p* = 0.066). A paired t-test was used to compare each group’s cycles to failure, load at failure, and dynamic stiffness (*α* = 0.05).

## Results

**Figure 2:**
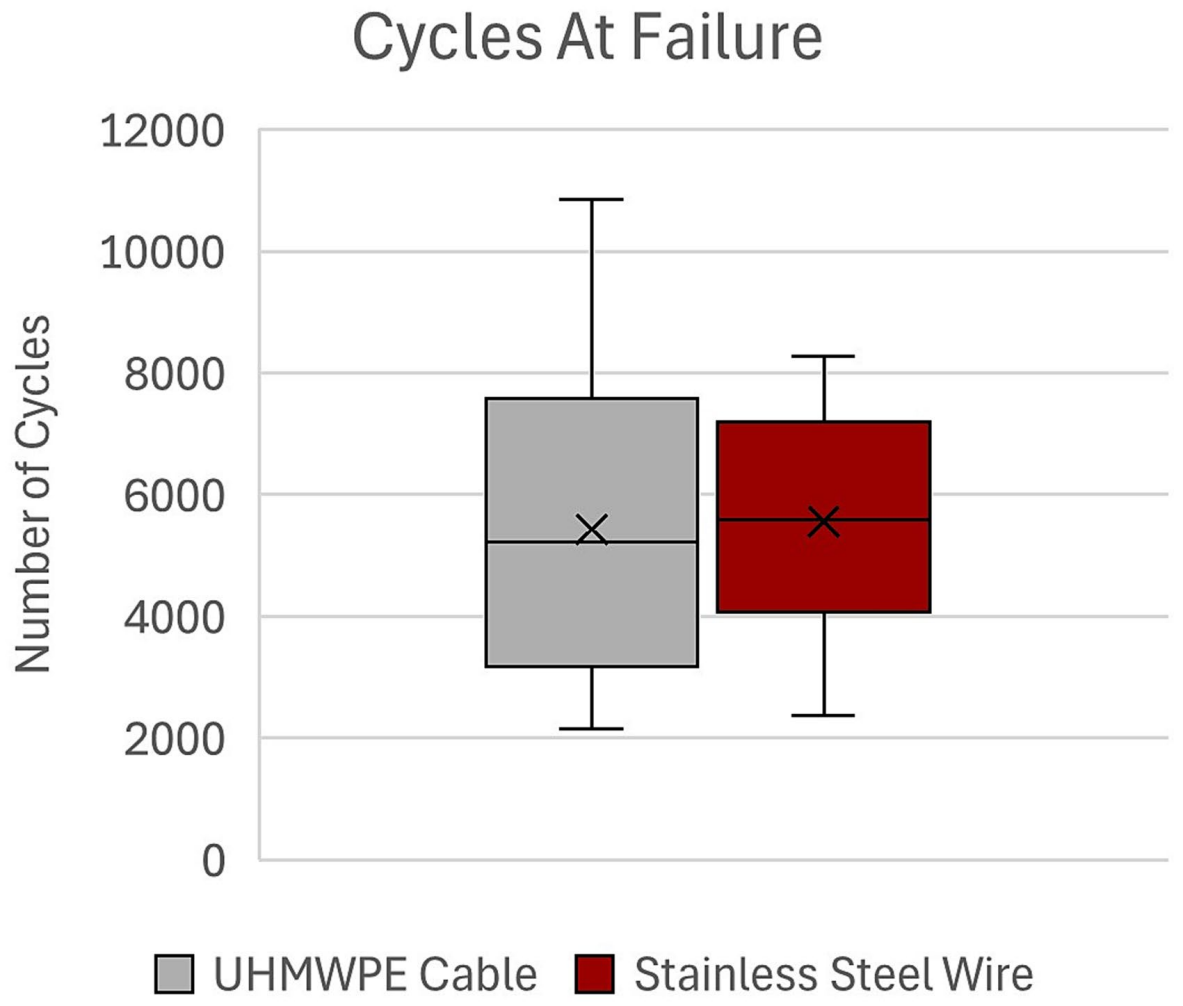
Cycles at failure of UHMWPE cable and stainless steel wire constructs. UHMWPE, ultra-high molecular weight polyethylene; SSW, stainless steel wire.

**Figure 3:**
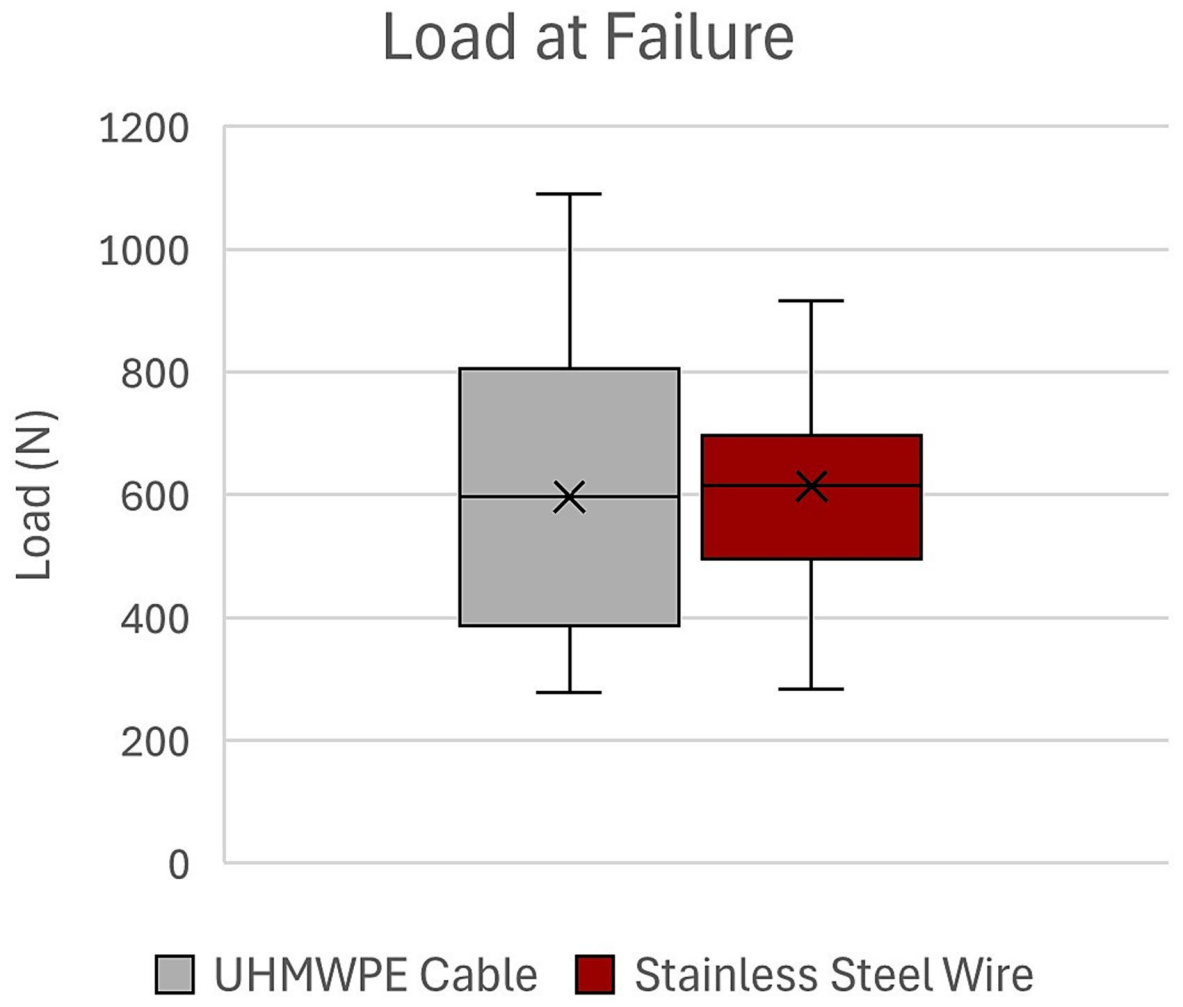
Load at failure of UHMWPE cable and stainless steel wire constructs. UHMWPE, ultra-high molecular weight polyethylene; SSW, stainless steel wire.

**Figure 4:**
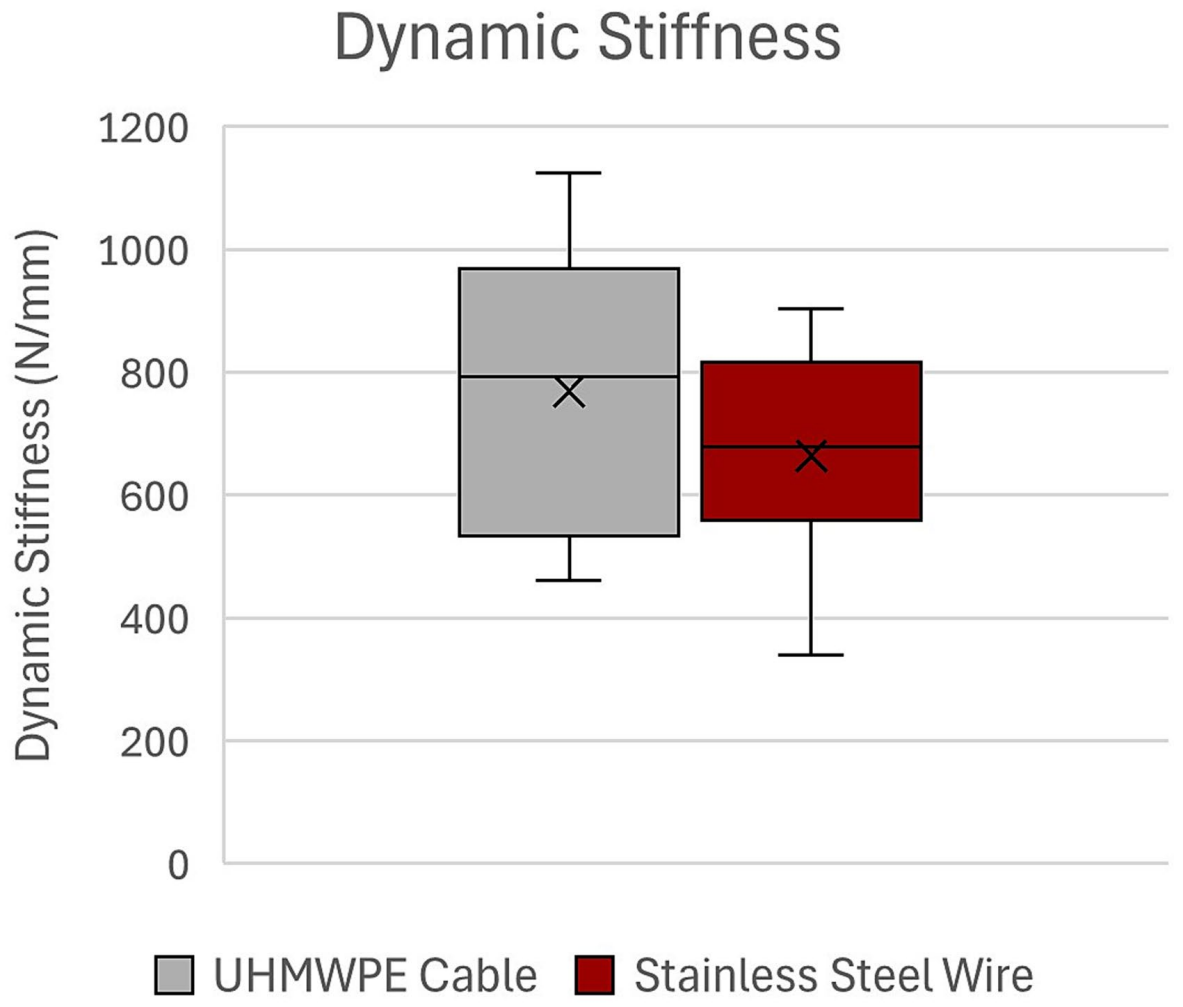
Dynamic stiffness of UHMWPE cable and stainless steel wire constructs. UHMWPE, ultra-high molecular weight polyethylene; SSW, stainless steel wire.

There was no statistically significant difference in number of cycles to failure for UHMWPE (5,430 ± 2,775) as compared to SSW (5,558 ± 1884) (*p* = 0.898) (Figure 2). Load at failure for UHMWPE (597.4 ± 268 N) and SSW (614.2 ± 198.5 N) was not statistically significant (*p* = 0.858) (Figure 3). Additionally, there was no statistically significant difference in mean dynamic stiffness of UHMWPE (768.8 ± 232 N/mm) and SSW (663.6 ± 170.6 N/mm) (*p* = 0.167) (Figure 4). High variability was observed in the paired differences for all outcome variables (cycles to failure SD = 3077.3; load at failure SD = 288.5; dynamic stiffness SD = 221.4). No breakage or fraying of cable or failure at clamps of the UHMWPE cables were noted during this study. The UHMWPE cable constructs all failed by permanent lateral displacement of each fracture fragment’s ends without obvious cable loosening. No breakage of metallic wire was observed in any construct; however, permanent deformation of the wire was noted in 2 of the 10 constructs. Visible wire loosening was noted in all constructs using metallic wire after cyclic loading following the permanent lateral displacement of the fracture fragment ends.

## Discussion

This study compared the mechanical performance of an UHMWPE orthopedic cable system to a similarly sized stainless steel loop cerclage under cyclic four-point bending. Under these conditions, there was no statistically significant difference in cycles to failure, load at failure, or dynamic stiffness of the UHMWPE cable and SSW constructs and the author’s hypothesis was rejected. The lack of a statistically significant difference could be due to equivalence between the fixation modes in our study design. However, a clinically and statistically significant difference may exist between the fixation methods that our study was not adequately powered to detect, particularly given the limitations of an in-vitro study setting. Despite the study’s limitations and lack of statistical significance, our findings suggest that UHMWPE cable has comparable biomechanical stability to SSW placed using the single loop cerclage method.

Metallic stainless steel orthopedic wire is commonly used in veterinary orthopedic surgery. It is widely available, relatively economical, and its mechanical properties are well studied. As with any implant, it is important to consider its properties and limitations during implant selection. SSW is strong in resisting forces aligned with the wire but is susceptible to low-cycle fatigue failure (7). One distinct disadvantage is the risk of needing revision surgery or implant removal when complications such as soft tissue irritation, fixation loss due to wire loosening or breakage, infection, osteolysis, and implant associated pain occur (9–11).

Multifilament metallic cerclage is another alternative to monofilament SSW cerclage. A few isolated case reports exist of metallic cerclage being used to treat revision total hip arthroplasty and stabilization of a periprosthetic femoral fracture in a dog. Cable cerclage use was described by Blaeser et al. as part of a successful revision procedure following aseptic loosening of a total hip arthroplasty femoral implant (18). A stainless-steel orthopedic cable-plate system was also described to successfully stabilize a periprosthetic femoral fracture in a dog following total hip arthroplasty (19). In both studies the cable cerclage was used to salvage total hip arthroplasty complications successfully. However, failure of multifilament cerclage cable systems has historically been a concern precluding their use in clinical cases. The main concerns include reported high failure rate leading to post-operative complications and loosening during surgery (13, 20). Metallic cables are noted to undergo fatigue failure and fray which can potentially release metal particulate into the body in clinical applications (13). In contrast, UHMWPE cables did not show any visible damage under cross examination and examination under scanning electron microscopy after 1 million loading cycles (21). Resilience of implants to resist damage over many cycles is particularly important in veterinary patients as control of post-operative patient mobility is more limited as compared to human patients. No breaking, fraying or observable loosening of cable or failure at clamps were noted during the present study. No breakage of metallic wire was observed; however, mild observable permanent deformation was noted in two stainless steel cerclage constructs, and loosening of wire was noted in all constructs using metallic wire after cyclic loading.

Loop cerclage wire is tensioned using a wire tightener device. Drawbacks to this technique are that the correct tension is applied by surgeons’ experience and its “feel.” The desired tension for application may not be sustained if there is excessive motion during placement or cutting of the wire following tensioning. Conversely, appropriate tension may be under-applied resulting in loosening of the implant prematurely. Surgeon experience does not seem to affect tension of applied wire (1). With the UHMWPE cable system, a tensioner provides a method of applying tension to the cable to achieve 80 or 120 lbs. This theoretically helps standardize the level of tension applied to the cable during implant application. Ménard et al. tested a variety of multifilament orthopedic cables and noted loss of tension at crimping and following removal of the tensioner device of all cables tested (20). This included UHMWPE cable. This may warrant further investigation and stresses that proper technique and consideration of the tension required for the intended purpose of the cable is important regardless of the choice of metallic versus non-metallic cable used.

There are relatively few studies evaluating non-metallic cables in comparison to SSW in the veterinary literature, but the results of existing studies suggest that non-metallic wire may be a valid alternative in certain applications. Specifically, Hwang et al. investigated the biomechanical properties of a non-metallic cable (FiberWire) as compared to a pin and tension band technique using a canine cadaveric olecranon fracture model. This study found that the mean maximum load and yield load were higher for the non-metallic cable than that of metal wire (15). Additionally, Rothaug et al. found that the ultimate tensile strength of another non-metallic cable (Secure Strand) was greater than SSW when used to repair simulated midbody sesamoid fractures in an equine cadaveric model (14).

The use of nonmetallic cables in human surgical practice is well-documented. Non-metallic wire has been demonstrated to be biomechanically similar in strength with potentially fewer reported complications (9–12). In a retrospective study of 29 patients, the UHMWPE was used to augment primary and revision total hip arthroplasty surgeries. It was concluded that the nonmetallic periprosthetic cables provided adequate fixation to allow for osteotomy and fracture healing with no complications related to the cables themselves (22). The UHMWPE is preferred by some human surgeons performing pin and tension band repairs of olecranon avulsion fractures. In a case series of 7 patients with olecranon fractures, the UMHWPE cable maintained anatomic reduction of fractures through bone union and yielded excellent physical and functional outcomes (23). Individual reports where the UHMWPE cable was chosen as part of the fixation include a scapulothoracic fusion (24), revision shoulder arthroplasties (25), and repair of periprosthetic femur fractures (26).

Infection remains a major concern affecting all orthopedic implants and bacterial adherence is an important consideration for implant selection. Multifilament suture material has been shown to have decreased ability to resist infection as compared to their monofilament counterparts (27). Masini et al. investigated the bacterial adherence of several multifilament high-tensile strength suture materials and showed that it varies significantly between the materials tested (28). The UHMWPE cable evaluated in the present study has been shown to have decreased biofilm formation compared to metallic wire in *in vitro* conditions (29). Further investigation is warranted on bacterial adherence and risk of infection regarding this implant *in vivo*.

There are several limitations noted in the present study. Firstly, there are inherent limitations to simulating in vivo physiologic forces using cadaveric models. All soft tissue was stripped from the cadaveric bones to allow embedding within polyurethane casting resin for biomechanical testing. As such there is no way to evaluate the impact of soft tissue on either implant application or contributions to overall stabilization of the model. The authors also note that cerclage is traditionally used with other primary modes of fixation and not typically used alone. No ancillary fixation was used in this study in order to mechanically isolate the implants tested. Additionally, previous biomechanical studies have established that the addition of intramedullary pinning was not significantly protective of loop cerclage fixation for a diaphyseal long-oblique fracture model under four-point bending (5). Furthermore, a fracture repair would be subjected to a multitude of forces in the clinical setting. The present study did not aim to describe all biomechanical forces acting on a fracture, nor to simulate a femoral fracture repair, but rather to highlight the mechanical differences of the implants tested in a single mode of loading using a standardized model. Additionally, the forces applied during testing in this study far exceeds the forces a canine femur would likely experience during the convalescent period in a normal dog. Although exact measures of force on the femur *in vivo* are difficult to ascertain, three dimensional models estimate the average hip joint reaction force on the femur of a mixed breed 23 kg dog to be 238.78 N or 1.04 times its body weight during a three-legged stance (17, 30). Analysis of ground reaction forces of the hind limb of dogs at a trot generated forces up to 74.04 percent of body weight in medium breed dogs (31). Use of a plate, pin or external fixation to mimic clinical applications under physiologic axial load should be considered for future biomechanical studies.

This study successfully achieved its objective of determining variability and estimating differences between groups. Specifically, the high variability observed in the outcome variables provides valuable information that future researchers can use to design adequately powered studies; larger sample sizes will be required to detect statistically significant differences between groups. All implants were applied by the same investigator (SD), but this still resulted in a large amount of variability. Sources of variability to consider include physical variability of specimens or potential inconsistency during implant placement. Operator variability may be an important source of inconsistency especially in consideration of the significant loss of tension at clamping and during removal of the tensioner found in Menard et al.

In conclusion, this study supports previous findings that non-metallic cables represent a promising alternative to SSW cerclage. Future clinical studies are needed to assess how UHMWPE cables perform *in vivo* in veterinary patients.

## Data Availability

The raw data supporting the conclusions of this article will be made available by the authors, without undue reservation.

## Footnotes

^1^SuperCable Iso-Elastic™ Cerclage Cables, Kinamed Inc., Camrillo, California, United States.
